# Effects of Removable Functional Appliances on the Dentoalveolar Unit in Growing Patients

**DOI:** 10.3390/medicina60050746

**Published:** 2024-04-30

**Authors:** Filippo Cardarelli, Sara Drago, Luigi Rizzi, Martina Bazzani, Paolo Pesce, Maria Menini, Marco Migliorati

**Affiliations:** 1 Interdisciplinary Department of Medicine, University of Bari “Aldo Moro”, 70121 Bari, Italy; drfilippocardarelli@libero.it; 2 Department of Surgical Sciences and Integrated Diagnostics (DISC), Genova University, 16132 Genoa, Italy; dr.sara.drago@gmail.com (S.D.); paolo.pesce@unige.it (P.P.); maria.menini@unige.it (M.M.); 3 School of Dentistry, Genova University, 16126 Genoa, Italy; dr.rizzi.luigi@gmail.com (L.R.); m.bazzani001@gmail.com (M.B.)

**Keywords:** orthodontics, class II, early treatment, elastodontic

## Abstract

*Background and Objectives:* The objective of this retrospective controlled study is to compare class II growing patients who underwent treatment with two different functional appliances: the Fraenkel regulator (FR-2), utilized as the control group, and the elastodontic device “Cranium Occluded Postural Multifunctional Harmonizers” (AMCOP), utilized as the test group. *Materials and Methods:* The study sample consisted of 52 patients with class II division I malocclusion (30 males, 22 females, mean age 8.6 ± 1.4 years) who were treated with the two different types of appliances: Group 1 (*n* = 27, mean age 8 [7.00, 9.00] years, 12 females, 15 males) received treatment with AMCOP, while Group 2 (*n* = 25, mean age 9.2 years [8.20, 10.00], 10 females, 15 males) received treatment with FR-2. The mean treatment duration for Group 1 was 28.00 [21.50, 38.00] months, while for Group 2 it was 23.70 [17.80, 27.40] months. Cephalometric analyses were performed on lateral cephalograms taken before treatment (T1) and after treatment (T2). *Results:* Significant intragroup differences were observed over time in Group 1 for 1^/PP. Similarly, significant intragroup differences were observed over time in Group 2 for SNB, ANB, and IMPA. *Conclusions:* Both treatment modalities resulted in the correction of class II malocclusion with dentoalveolar compensation, although the treatment duration with AMCOP tended to be longer on average.

## 1. Introduction

Functional appliances are a large and diverse family of orthodontic appliances; they were introduced in the early 1900s in Europe and since then have gained popularity all over the world. 

Functional devices encompass a variety of active or passive appliances designed to facilitate the proper growth of bone bases by influencing the orofacial musculature. These appliances mimic perioral muscles, transmitting forces to both the teeth and basal bones, thereby inducing orthodontic and orthopedic changes. Early intervention to address any dentoskeletal malocclusions or muscular interferences at a very young age leads to superior results and ensures a stable balance between the jaws and muscular components. The inherent skeletal plasticity of preschool-aged children allows for swift and enduring therapy outcomes, guaranteeing long-term stability [1].

Functional appliances can either be removable or fixed. Over the years, numerous types of functional devices have been developed, usually bearing the name of their inventor: Bimler, Fränkel, Planas, Cervera, Bass, and Twin Block [2,3,4]. Functional appliances all have a postural effect on the mandible, although how this is achieved and the auxiliary components they incorporate varies between different systems [5].

The main problem with removable functional appliances is compliance. These are often difficult appliances to wear as they can affect speech and oral function and therefore not all patients tolerate them. From prospective studies, failure rates have been reported of up to 34% for Twin Blocks. This is primarily due to non-compliance. Fixed functional appliances theoretically remove the problem of cooperation but are more prone to breakage and are more expensive [6]. 

The Class II, division 1 malocclusions have long been noted as the most frequent treatment problem in orthodontic practice [7] and may lead to an increased risk of incisor trauma, teasing, and bullying [8,9]. It may also have a negative impact on oral health-related quality of life [10]. 

Many treatment approaches are currently available to orthodontists and the solution can involve the use of functional or fixed orthodontic appliances, or both [11,12,13,14]. 

Among the fixed functional appliances (FFA), the Herbst, proposed by Emil Herbst [15] and Hans Pancherz [16], shows effects on Class II patients that are both dental and skeletal. From a dental point of view, they produce a reduction of the overjet and different effects on the mandibular and maxillary arch: while the first undergoes an anterior displacement, the upper arch shows a posterior displacement. Skeletally, the effects include enhanced forward position of the mandible and reduced growth of the maxilla [17]. Nevertheless, side effects have been reported from previously published articles due to anchorage loss: dental anchorage loss produces side effects on the final incisor position, both upper and lower, as well as the uncontrolled movement of posterior teeth. Mandibular incisors’ proclination and maxillary incisors’ retroclination negatively affect the Herbst appliance effect, because of the reduction of mandibular advancement space [18]. These side effects can be limited using a total of four miniscrews inserted in the mandibular and maxillary arch as anchorage reinforcement [19]. 

McNamara maintained that the most frequent skeletal problem in Class II patients is mandibular retrognathia [20]. This suggests that the use of appliances with a demonstrated ability to stimulate mandibular growth may be the solution to this problem. For these patients, therapy for stimulating mandibular growth by means of forward positioning of the mandible, provided by functional appliances, is indicated. Furthermore, functional appliances transmit muscular forces to the dentition and alveolus stimulating mandibular growth by means of forward positioning of the mandible to alter a Class II relationship. 

One of the most popular among the functional appliances is the function regulator (FR-2), proposed in the early 1960s by Fränkel [21,22]. This appliance is designed to induce mandibular advancement and facilitate skeletal correction by harnessing the natural growth potential of the mandible. The FR-2 appliance was designed by Fränkel as an exercise device used to correct the function of the circumoral musculature. 

The evolution of materials has led to the introduction of new preformed appliances known as elastomeric devices. Elastomeric devices represent a specific category of interceptive and functional appliances, distinguished by their simplicity of use for patients, safety, and construction. Additionally, these devices are crafted from a soft material that minimizes the risk of damage to oral soft and hard tissues during usage. Their design promotes ease of use and ensures patient comfort throughout treatment, making them a preferred option in contemporary orthodontic practice. Numerous types of elastodontic devices exist: “Cranium Occluded Postural Multifunctional Harmonizers” (AMCOP, Micerium spa) is one elastodontic approach available on the market. These appliances are composed of two flanges, one vestibular and one lingual, which delimit a free central area. The aim of these devices is to stimulate groups of muscles working on the positions of the mandible, tongue, and dental arches by applying light and biological forces. Vestibular flanges aim to stop the influence of perioral muscles on the teeth and jaw position; meanwhile, the elastomeric material and the neuromuscular system cooperate to gain the desired tooth position [23,24,25]. 

The purpose of this retrospective control study is to compare patients treated with two different functional appliances: the Fraenkel regulator (FR-2) used as the control group, and the new elastodontic device, AMCOP, as the test group. This study aims to evaluate the effectiveness of these two appliances in achieving desired treatment outcomes and to provide valuable insights into their respective roles in orthodontic therapy.

## 2. Materials and Methods

### 2.1. Subject

In this retrospective study, a total of 52 patients, categorized into two groups, were subjected to analysis. Group 1 (*n* = 27, mean age 8 [7.00, 9.00] years, 12 Females, 15 Males) underwent treatment with AMCOP, whereas Group 2 (*n* = 25, mean age 9.2 years [8.20, 10.00], 10 females, 15 males) received treatment with FR-2. The mean treatment duration for Group 1 was 28.00 [21.50, 38.00] months, while Group 2 had a mean treatment duration of 23.70 [17.80, 27.40] months.

All the treatments were conducted at the orthodontic department of the University of XX. All measurements were conducted by the same operator using OnyxCeph software (Version 3.2.63, Image Instruments GmbH, Chemnitz, Germany). A blinded statistical analysis was performed to maintain objectivity and accuracy in the interpretation of the results.

### 2.2. Inclusion Criteria

In order to be considered for participation in this study, patients needed to meet specific criteria, including presenting with a class II division I malocclusion characterized by an overjet exceeding 5 mm and a complete class II molar relationship. Additionally, they were required to be in good general health and have no history of previous orthodontic treatment.

### 2.3. Devices

Elastodontics is a form of orthodontic treatment that utilizes removable devices made of elastomeric material. These devices are user-friendly, safe, and easy to use for patients, and are used for interceptive treatment [26]. The first elastodontic device was presented by Kesling in 1945: it was meant to be a finishing device or a contention after multibracket therapy that did not require an impression nor the creation of a setup [27,28]. Moving on with the evolution, the first elastodontic functional device, known as an “activator”, was presented in France by Soulet and Besombes in 1950. The idea behind this device was that the material of which the device was made allowed for the induction of skeletal and neuromuscular effects, alongside the repositioning of the mandible and the maxilla within the cranial system [29,30]. 

Currently, many different elastodontic devices are available in the literature and on the market. In our research, we decided to study the AMCOP (Micerium, Avegno, Italy) bioactivator designed for class II correction. 

The Bioactivators consist of two flanges, positioned on both the vestibular and lingual sides, thereby creating a central open area. This design feature, devoid of indents, facilitates the simultaneous treatment of both dental arches, employing multidimensional orthopedic repositioning to allow teeth to move freely without constraints. Moreover, these devices serve as a therapeutic option for rectifying altered muscle function. The lingual ramp and tongue button further contribute to restoring proper posture and lingual function. Different types of these devices are available, each tailored to address specific malocclusions, distinguished by varying colors and transverse sizes. Patients underwent treatment with either a single bioactivator or two distinct Bioactivators, depending on treatment requirements. All patients received treatment with a second-class (SC) bioactivator, featuring a mandibular anterior sliding plane to facilitate mandibular advancement, correct overjet through retro inclination of upper incisors, proinclination of lower incisors, and enhancement of TMJ function. Regular checks were conducted to monitor progress and make any necessary adjustments [24,25,26].

Functional posterior crossbites can be effectively treated using elastodontic devices [31]. In cases where upper arch expansion was deemed necessary, a First Class Integral bioactivator of type S was employed prior to the SC bioactivator. The Type S bioactivator is specifically designed for use in patients with a mesocephalic cranial index and oval dental arches. It is important to note that patients treated with other types of AMCOP bioactivators were not included in the study, underscoring the specificity of the treatment approach.

The Fraenkel appliance is a functional appliance that works by exerting pressure on soft tissues [32]. 

The appliance design prioritizes minimal contact with the teeth, aiming for tooth movement via soft tissue manipulation rather than direct tooth movement. Its construction emphasizes locating most of the structure in the vestibule to separate the lips and cheeks from the teeth, promoting arch expansion and jaw growth. Featuring large buccal shields and lip pads, the Fraenkel appliance reduces pressure on the dentition from the cheeks and lips, facilitating maxillary arch expansion and correction of Class II malocclusions [33]. Additionally, it includes a small lower lingual pad behind the lower incisors to advance the mandible forward, and a facial pad in front of the lower incisors to hold the lower lip forward. Despite its perceived bulkiness, much of the appliance’s structure resides in the buccal vestibule. The presence of the pad in the vestibular area aids in repositioning the lower lip correctly, stimulating normal mandibular growth and positioning. Gradual activation of the lingual pad and lip shields by anterior movement corresponds to mandibular growth [3].

### 2.4. Sample Size

The sample size estimation, calculated based on statistical parameters, determined that a minimum of 25 patients per group would be required to achieve 80% power in detecting a mean difference of means in the ANB parameter of 1.6°. This calculation factored in an assumed standard deviation of differences of 2° and a significance level (alpha) of 0.05 using a *t*-test, ensuring robustness in the analysis methodology.

### 2.5. Statistical Analysis

Data were tested for normality with the Shapiro-Wilk test. Continuous variables are given as means ± standard deviations (SD) and medians with interquartile range, whereas categorical variables as numbers and/or percentages of subjects.

Baseline differences between groups were tested using the student’s *t*-test, Mann–Whitney U test, or Fisher’s exact test. Intragroup differences over time were tested by the paired *t*-test or Wilcoxon’s signed rank test. To investigate the association of differences over time with groups, the student’s *t*-test and Mann–Whitney U test were performed again. Differences with a *p*-value < 0.05 were selected as significant. Data were acquired and analyzed in the R v4.2.2 software environment.

ICC was measured for both linear and angular values and values were 0.81 and 0.91 respectively.

### 2.6. Treatment Protocol

GROUP 1: The AMCOP devices were utilized for approximately one hour each day and throughout the entire night for the initial six to eight months, after which they were exclusively worn during nighttime hours. In instances of second-class malocclusion with severe contraction of the upper arch (bilateral crossbite), treatment commenced with the “AMCOP Integral” to rectify the correct transverse diameter, followed by subsequent use of the “AMCOP SC” solely during nighttime to address sagittal concerns. Alternatively, in cases of mild upper arch contraction, intervention with the “AMCOP SC” directly was preferred to expand the upper arch, facilitate mandibular protrusion, and harmonize arch coordination. Selection of devices was guided by cephalometric analysis, while the utilization of chewing planes and various arch forms enabled targeting of diverse malocclusions primarily based on transverse and vertical dimensions before addressing sagittal concerns. In numerous cases, optimal mandibular positioning was achieved only after upper arch expansion, whereas in others, mandibular advancement followed resolution of transverse issues, facilitated by the specific device, the “AMCOP SC”. Modifications were limited to adjustments in the bioactivator length; for cases requiring expansion, larger and longer devices were chosen, necessitating a reduction in posterior length to prevent interference in distal zones. In select cases, slight reductions in the vestibular shield measure were made to prevent interference with a short upper lip frenulum. Monthly checks were conducted to monitor progress [24,25,26]. 

GROUP 2: Patients were directed to consistently wear the FR-2 device for a minimum duration of 16 h per day, encompassing both nighttime and daytime wear, with the exception of school hours, for the entire duration of the treatment protocol. Throughout the treatment regimen, lingual and lip shields were adjusted in accordance with the sagittal movement of the mandible to address any transverse deficiencies that may arise. Each device was meticulously tailored based on individualized cephalometric analysis, ensuring precise customization for each patient’s needs. The incorporation of chewing planes and diverse arch forms facilitated the targeted correction of various malocclusions, encompassing transverse, vertical, and sagittal dimensions.

### 2.7. Cephalometric Analysis

Cephalometric Analyses were meticulously conducted on lateral cephalograms taken prior to the commencement of treatment (T1) and subsequent to the completion of treatment (T2). These comprehensive analyses were meticulously performed using the sophisticated Onyx Ceph software by a single proficient operator. Subsequently, to ensure the utmost accuracy and reliability of the results, a second experienced operator meticulously validated the cephalometric analyses. The analysis encompassed a total of ten angular measurements meticulously aimed at evaluating cephalometric changes pre- and post-treatment. The assessments focused on orthopedic changes in both the sagittal and vertical planes, alongside meticulously tracking dental adjustments.

## 3. Results

A total of 52 patients (30 M, 22 F, mean age 8.6 ± 1.4 years) were analyzed in this study. Of these, 27 patients received treatment with a bioactivator (Group 1), and 25 patients were treated with a Fraenkel appliance (Group 2).

Age at the beginning of treatment, and therapy duration, showed a significant difference between groups (*p* = 0.027 and *p* = 0.021, respectively). 

No significant differences were found at baseline, except for ANB (*p* = 0.038), 1^/1^ (*p* = 0.049), and 1^/PP (*p* < 0.001), as reported in Table 1.

Significant intragroup differences were found over time in Group 1 for 1^/PP (*p* = 0.042).

Significant intragroup differences were found over time in Group 2 for SNB (*p* = 0.023), ANB (*p* < 0.001), and IMPA (*p* = 0.018); see Table 2.

No significant differences were found between groups regarding longitudinal changes for any parameter.

## 4. Discussion

This is a retrospective study comparing two different treatment protocols of class II division I malocclusion in growing patients: the Frankel II appliance and AMCOP bioactivator.

Class II division I treatment is one of the most common problems to be solved by an orthodontist, and many appliances have been proposed in the literature, including fixed functional appliances such as the Herbst Appliance, the Forsus Fatigue Resistant device, MARA, and many others. All of these FFA could successfully achieve class II correction even though patient discomfort and difficulties in daily clinic management may reduce their use. Removable functional appliances (RFA) have an extensive literature background with many advantages for the patient and few side effects; compliance remains the greatest problem with RFA that can ultimately lead to unfavorable results [34,35,36,37,38,39]. 

AMCOP bioactivators may be a valid alternative for the treatment of growing patients. According to Inchingolo et al. [25], AMCOP bioactivators present different advantages compared to traditional functional devices. These devices are easy to use for both the patient and the clinician and they are safe. The instructions given to patients on these devices were to wear the appliance for a few hours a day; for this reason, better compliance of the patient can be achieved. Thus, AMCOP could result in a less invasive functional device. Since there is no need for a lab to prepare the device, costs are lower and there is no need for an intraoral scan or impression. The intermolar distance is used to determine the proper appliance size. Furthermore, these devices are made of soft materials that do not cause damage to soft tissues. Moreover, since elastomeric equipment acts as a shield that isolates the dentoalveolar structures from perioral muscles, previous evidence would suggest that it is possible to achieve the rebalancing of the perioral musculature similar to the one obtainable with rigid functional equipment such as a Fränkel appliance. The market for elastodontic appliances is continuously growing and offers a plethora of devices; however, this is in contrast with the lack of adequate scientific evidence validating their clinical usage. A literature review via PubMed based on the term “AMCOP” only gives four results in a time span of the last four years: three clinical [23,24,25] studies and one in vitro [40] study.

According to Fränkel [21,22], a normal pattern of muscular behavior fosters normal skeletal and dental development, sustaining the newly established mandibular position. The FR-2 appliance is distinguished by its projecting vestibular shields, which promote expansion of the orofacial muscles and facilitate an anterior functional shift of the mandible. This functional shift is instrumental in achieving favorable skeletal and dental alignment, as advocated by Fränkel’s research [21,22]. 

As stated by Petrovic et al. in their studies, the Fränkel appliance does not act by a lower teeth mesialization, although there might be some premolar and molar migration, but by stimulating the growth rate and the amount of condylar cartilage and increasing the subperiosteal growth rate at the mandibular ramus [41,42]. This has also been confirmed by Cevidanes et al. in their prospective studies which showed that treatment with FR-2 appliances promoted changes in maxillary and mandibular position, affecting ramus alignment and ramus vertical dimensions relative to the middle cranial fossae and posterior nasomaxilla dimension [43]. On the other hand, Chadwick et al. found that the functional regulator of Fränkel does not produce any statistically significant skeletal changes [44]. Instead, another study stated that the skeletal changes are due to a limitation in maxillary growth more than an enhancement in mandibular growth [45]. 

Despite the controversy about the skeletal effects, the Frankel appliance’s effectiveness in dental changes is well known. As reported in several articles, the effect of the FR-2 appliance is to procline the lower incisors while maxillary incisors get tipped lingually [46]. This statement is supported by literature, observing that FR-2 is also known for its favorable and stable results over the long term with both skeletal and dentoalveolar changes [47].

In the present study, both appliances showed a slight improvement in sagittal skeletal values; the amount of correction is similar in absolute value even though the FR-2 appliance showed statistically significant differences. Nevertheless, the overall skeletal correction can be compared to normal mandibular and maxillary growth with slightly more mandibular advancement when FR-2 is used.

Vertical skeletal changes were similar to other reports where no statistically significant clinical changes were observed. Both appliances showed similar effects both on mandibular vertical position and mandibular angle.

Assessment of the sample demographics and cephalometric parameters indicated that the data of patients treated with either the AMCOP bioactivator or the Frankel FR-2 were clinically comparable at baseline (Table 1). In our study, both groups exhibited a similar distribution of males and females, with slightly younger ages observed in patients treated with the AMCOP group. This parity in baseline characteristics ensures a robust comparison between the two treatment modalities and enhances the validity of our findings.

Measurements in Group 1 reported a statistically significant difference in upper incisor inclination (1^/PP Group 1: −3.00 [−6.00, 1.50]). There were no other statistically significant differences.

Measurements in the control group may support previous literature evidence: treatment resulted in a lower incisors proclination and an upper incisors retroclination. Interestingly, lower incisors proclination values were limited in both groups, showing a positive control of the lower anterior teeth during treatment avoiding excessive buccal movements. The control group presented, according to the Frankel literature, statistically significant skeletal changes in mandible growth and a reduction of the ANB angle, even though those values could not be considered clinically significant. For the test group, the skeletal changes were not significant despite being similar to Frankel’s change values.

Inter-incisor angle variations were similar between Group 1 and another group reported in the literature by Fichera et al. [23], although in our study the variation was not statistically significant.

Both treatments led to the resolution of the class II malocclusion but treatment duration for AMCOP was longer on average.

### Study Limitations

Retrospective design of the study represents the main limitation of the study. Prospective studies as well as randomized clinical trials can more accurately describe effects in growing class II patients. Retrospective study could lead to potential selection bias of treated patients, although in this case only consecutively treated patients were included in the analysis. Further prospective studies are needed to evaluate mandibular advancement values and even the maxillary transverse dimensional changes. 

Further analysis should include practice management aspects, such as chair-side time, overall costs, and patient satisfaction surveys.

## 5. Conclusions

Considering the outcomes derived from the present study, the ensuing conclusions can be summarized as follows: AMCOP and FR-2 devices were found to be efficacious in treating Class II division I malocclusion with dentoalveolar compensation. AMCOP treatment led to greater dental compensation over a slightly prolonged period of time, as evidenced by the results of the study.

## Figures and Tables

**Table 1:** Demographic and baseline characteristics of groups. Results are expressed as Mean ± Standard Deviation or Median [Interquartile Range]; Intergroup *p*-value = student’s *t*-test *p*-value adjusted using Bonferroni method or Mann–Whitney U test, *p*-value adjusted using Bonferroni method or Fisher’s exact test (percentages) *p*-value. Cephalometric parameters: SNA: angle formed between point A, Sella, and nasion; SNB: angle formed between point B, Sella, and nasion; ANB: angle formed between point A, nasion and point B; NSBa: angle formed between nasion, Sella and Basion; FH^PP: angle formed between Frankfurt plane and palatal plane; FMA: angle formed between Frankfurt plane and mandibular plane; Ar-Go-Me: gonial angle; 1^/1^: interincisal angle; 1^/PP: inclination of upper incisors on palatal plane; IMPA: inclination of lower incisors on mandibular plane. * Differences selected as significant (*p*-value < 0.05).

	Group 1 AMCOP	Group 2 Fraenkel	*p*-Value
*n*	27	25	
Age (years)	8.00 [7.00, 9.00]	9.20 [8.20, 10.00]	0.027 *
Sex			
F (%)	12 (44.4)	10 (40.0)	0.727
M (%)	15 (55.6)	15 (60.0)	
Therapy duration (months)	28.00 [21.50, 38.00]	23.70 [17.80, 27.40]	0.021 *
Cephalometric parameters			
SNA [°]	82.03 ± 3.20	81.82 ± 3.43	0.821
SNB [°]	77.12 ± 3.48	75.68 ± 3.37	0.137
ANB [°]	4.91 ± 2.18	6.14 ± 1.94	0.038 *
NSBa [°]	132.16 ± 4.44	130.50 ± 4.61	0.191
FH^PP [°]	−3.00 [−4.50, −2.00]	−2.00 [−3.50, −1.00]	0.188
FMA [°]	21.58 ± 3.88	21.88 ± 5.15	0.811
Ar-Go-Me [°]	124.04 ± 5.62	122.76 ± 4.16	0.359
1^/1^ [°]	128.87 ± 11.72	121.25 ± 15.21	0.049 *
1^/PP [°]	102.90 ± 8.33	115.52 ± 10.56	<0.001 *
IMPA [°]	96.43 ± 7.71	98.36 ± 9.05	0.411

**Table 2:** Differences over time in the whole population (*n* = 52). Results are expressed as Mean ± Standard Deviation or Median [Interquartile Range]; *p*-value: Intragroup *p*-value = paired *t*-test *p*-value adjusted using Bonferroni method or Wilcoxon’s signed rank test, *p*-value adjusted using Bonferroni method. Intergroup *p*-value = student’s *t*-test *p*-value adjusted using Bonferroni method or Mann–Whitney U test, *p*-value adjusted using Bonferroni method. * Differences selected as significant (*p*-value < 0.05).

	Group 1 AMCOP	Group 2 Fraenkel	Intergroup *p*-Value
*n*	27	25	
SNA [°] *p*-value	−0.20 [−0.65, 0.65] 0.791	0.00 [−1.50, 1.00] 0.169	0.345
SNB [°] *p*-value	0.50 [−0.50, 2.10] 0.16	1.00 [−0.50, 1.50] 0.023 *	0.633
ANB [°] *p*-value	−1.00 [−2.00, 0.70] 0.105	−1.00 [−2.00, −0.50] <0.001 *	0.741
NSBa [°] *p*-value	0.22 ± 3.26 0.73	−0.58 ± 2.93 0.331	0.358
FH^PP [°] *p*-value	0.93 ± 3.23 0.149	0.92 ± 2.28 0.055	0.994
FMA [°] *p*-value	−0.20 ± 2.43 0.672	0.48 ± 1.99 0.24	0.277
Ar-Go-Me [°] *p*-value	−0.48 ± 2.31 0.289	−0.70 ± 1.72 0.053	0.702
1^/1^ [°] *p*-value	1.00 [−4.50, 8.50] 0.14	1.50 [−1.50, 9.00] 0.389	0.735
1^/PP [°] *p*-value	−3.00 [−6.00, 1.50] 0.042 *	−1.00 [−7.50, 1.00] 0.088	0.905
IMPA [°] *p*-value	0.61 ± 5.18 0.548	2.90 ± 5.70 0.018 *	0.135

## Data Availability

The data that support the findings of this study are available from the corresponding author upon reasonable request.
